# Extracted Spent Coffee Grounds as a Performance-Enhancing Additive for Poly(Lactic Acid) Biodegradable Nursery Bags in Agriculture

**DOI:** 10.3390/polym17050561

**Published:** 2025-02-20

**Authors:** Amonrut Waisarikit, Nattawut Suadaung, Benjawan Khantho, Bawan Hadad, Gareth M. Ross, Paul D. Topham, Sukunya Ross, Sararat Mahasaranon

**Affiliations:** 1 Center of Excellence in Biomaterials, Department of Chemistry, Faculty of Science, Naresuan University, Phitsanulok 65000, Thailand; amonrutw62@nu.ac.th (A.W.); nattawut.su@nu.ac.th (N.S.); benjawan.kht@gmail.com (B.K.); gareth@nu.ac.th (G.M.R.); sukunyaj@nu.ac.th (S.R.); 2 Aston Institute for Membrane Excellence (AIME), Aston University, Birmingham B4 7ET, UK; 210110854@aston.ac.uk (B.H.); p.d.topham@aston.ac.uk (P.D.T.)

**Keywords:** spent coffee grounds, poly(lactic acid), agricultural bioplastic, green composite, nursery biodegradable bag

## Abstract

This study introduces biodegradable nursery bags using poly(lactic acid) (PLA), a widely used biodegradable polymer, and spent coffee grounds (SCGs), a byproduct of the brewing process in the coffee industry. SCGs were oil-extracted to produce extracted spent coffee grounds (exSCGs), which were characterized by their physical properties, chemical functionality, and thermal behavior. The exSCGs were blended with PLA at loadings of 5, 10, and 15 wt%. Analysis showed that exSCGs retained 3–5 wt% residual coffee oil, exhibiting a lower surface area (1.1163 m^2^/g) compared to SCGs (1.5010 m^2^/g), along with a higher pore volume (1.148 × 10^−3^ cm^3^/g) and pore size (~410 nm). All PLA/exSCG bio-composite films displayed a light brown color, well-dispersed exSCG particles, and excellent UV light barrier properties, with transmittance reduced to 1–2%. The residual coffee oil acted as a plasticizer, reducing the glass transition temperature, melting temperature, and crystallinity with increasing exSCG content. Mechanical testing revealed enhanced flexibility compared to neat PLA. Soil burial tests showed increased biodegradability with higher exSCG content, supported by SEM analysis revealing cracks around exSCG particles. The PLA/exSCG blend containing 10 wt% exSCGs exhibited optimal performance, with a significant increase in melt flow index (from 4.22 to 8.17 g/10 min) and approximately double the melt strength of neat PLA, balancing processability and mechanical properties. This innovation provides a sustainable alternative to plastic nursery bags, addressing waste valorization and promoting eco-friendly material development for agricultural applications.

## 1. Introduction

Plastic pollution has emerged as a critical global concern, with plastic particles now found in various environments, including soil, the deep sea, rainwater, and even within the human bloodstream and placenta [1,2,3]. To address this pressing issue, eco-friendly and recyclable materials are being increasingly integrated into product design. In agriculture, biodegradable alternatives, such as nursery bags, are gaining popularity for their potential to minimize microplastic contamination [4]. The agricultural grow bag or nursery bag market is projected to grow by USD 703 million between 2024 and 2030 [5]. Biodegradable nursery bags are particularly appealing due to their benefits for plant growth, such as promoting healthier root systems, regulating temperature, preventing overwatering, and efficiently degrading after use [6]. By decomposing naturally, these bags help reduce microplastic contamination in soil and water, minimize plastic waste, support environmental health, and mitigate wildlife impact [7].

Biodegradable films offer a promising solution in agriculture, particularly for nursery bags used to grow seedlings. Traditionally made from plastic, these bags protect young plants and create a controlled environment for root development. However, plastic nursery bags contribute significantly to pollution, as they can take hundreds of years to decompose. Biodegradable materials, such as poly(lactic acid) (PLA), poly(butylene succinate) (PBS), polycaprolactone (PCL), cellulose acetate butyrate (CAB), thermoplastic polyurethane (TPU), and thermoplastic starch, are ideal alternatives. These materials break down naturally through microbial activity in the soil after use, gaining popularity as sustainable solutions to replace traditional agricultural plastics [8,9,10,11,12,13,14]. Notably, PLA demonstrates impressive properties, holding the European Conformity composability trademark and FDA approval. However, its inherent brittleness limits its effectiveness in film applications. To enhance the physical properties of PLA, previous research has investigated blending it with other polymers or incorporating natural fillers and additives, such as rice husks, coconut coir, flax, clays, lemongrass, cassava starch, bamboo fibers, and cellulose nanofibers [14,15,16] The use of plant-friendly additives is highly advantageous for minimizing soil damage following product biodegradation. In this context, spent coffee grounds (SCGs) have emerged as a promising natural additive, offering an eco-friendly option that aligns with the goal of sustainable, biodegradable agricultural products in the present study.

Spent coffee grounds (SCGs) are byproducts of the brewing process in the coffee industry, accounting for up to 50% of the total cherry fruit waste. With coffee production projected to grow at an estimated rate of 4.37% per year between 2024 and 2028, the volume of SCG waste is also expected to increase significantly [17]. The utilization of SCGs has gained increasing importance. Numerous studies have investigated the potential applications of coffee industry waste, repurposing SCGs as a renewable energy source and for various other uses, such as in fertilizers, gardening, clean energy production, and mushroom cultivation [18,19,20]. The composition of SCGs includes polysaccharides, primarily hemicellulose and cellulose, as well as organic compounds such as fatty acids, amino acids, polyphenols, minerals, and melanoidins [21,22,23]. Consequently, SCGs hold significant potential as a natural filler in bio-composite materials. Various synthetic polymers and biopolymers have been used as matrix materials for composites incorporating SCGs, including high-density polyethylene (HDPE), polypropylene (PP), poly(butylene adipate terephthalate) (PBAT), poly(vinyl alcohol) (PVA), epoxy, and rubber composites [20,24,25,26,27,28].

Research conducted by Suaduang et al. previously explored the production of agricultural films using PLA incorporated with SCGs (particle size < 90 µm), examining the impact of SCG content on the physical and mechanical properties of bio-composite films produced with a twin screw extruder and a blow film extruder [29,30]. The results indicated that the incorporation of SCG particles significantly improved the flexibility of the films, suggesting that SCGs are an effective additive for creating more flexible PLA-based agricultural films. However, the ultra-high melt flow index (MFI) (>15 g/10 min) of the PLA/SCG bio-composite, attributed to the coffee oil content, as SCGs have been reported to contain approximately 16.7 to 17.2 wt% of coffee oil [31], limits their suitability for extrusion blow film processes at an industrial scale. This high MFI decreases the material processability, complicating consistent film production for large-scale applications.

Building on previous findings, the current study explores the potential of spent coffee grounds (SCGs) after oil extraction (resulting in exSCGs) as a natural additive to enhance the performance of biodegradable nursery bags. The primary goal is to optimize the formulation of PLA/exSCG bio-composites to improve both processing characteristics and product performance, addressing key challenges in the agricultural sector. Specifically, this work aims to enhance the feasibility of PLA/exSCG composites for large-scale applications by overcoming issues related to high melt flow index (MFI) and material processability.

This study takes an innovative approach to improving the processability of PLA-based biodegradable films. The research focuses on the controlled incorporation of exSCG particles to reduce the MFI and improve overall material properties. A key aspect of this work is the extraction of coffee oil from SCGs to create exSCGs, which lowers the oil content and directly addresses the high MFI associated with coffee oil. By removing excess oil without compromising the particle structure, this method improves the material’s rheological properties. Additionally, the particle size of exSCGs is reduced to below 45 µm, optimizing the composite’s performance during the extrusion blow film process. The smaller particle size enhances the interfacial interactions between the exSCG filler and the PLA polymer matrix, resulting in stronger, more processable composite materials. Together, these strategies of coffee oil extraction and particle size reduction are expected to significantly enhance the performance and processability of PLA/exSCG bio-composites, making them more suitable for the large-scale industrial production of biodegradable nursery bags. This approach not only improves the composite’s functionality but also advances the development of more sustainable materials for agricultural applications.

The processing of PLA-based bio-composite films has been explored using melt blending via extrusion, incorporating extracted spent coffee grounds (exSCGs) as an eco-friendly additive to enhance the flexibility and biodegradation rate of PLA films. The developed films and compounds were thoroughly characterized for their physical properties (appearance, morphology, and UV light transmission), rheological properties (MFI and capillary rheology), mechanical properties (tensile strength), and thermal properties (DSC and TGA). Furthermore, the potential of exSCGs as a natural filler to improve the economic viability and reduce the environmental impact of PLA/exSCG bio-composite film production has been assessed.

## 2. Materials and Methods

### 2.1. Materials

The natural filler used in this research, spent coffee grounds (SCGs), was collected from fresh coffee waste (Cafe Amazon, Phitsanulok, Thailand). Commercial-grade hexane was purchased from MODERN CHEMICAL Co., Ltd. (Bangkok, Thailand) Commercial-grade poly(lactic acid) (PLA, Ingeo 4043D) was purchased from BC Polymer Marketing Co., Ltd. (Bangkok, Thailand) (density = 1.24 g/cm^3^, melt flow index (MFI) = 6 g/10 min, melting point = 145–160 °C).

### 2.2. Preparation of the Extracted Spent Coffee Grounds (exSCGs)

Extracted spent coffee grounds (exSCGs) were produced from spent coffee grounds (SCGs). The spent coffee grounds (SCGs) were first thoroughly washed with deionized water three times to remove soluble compounds and contaminants. After washing, the SCGs were spread out in a thin layer and dried in a hot air oven at 60 °C for 12–16 h until they reached a low moisture content. The dried SCGs were then subjected to oil extraction using hexane at a 10:1 (hexane to SCG) ratio. The residual SCGs were ground into smaller particles using a grinder and sieve through a 325-mesh screen (particle size < 45 µm). Finally, the exSCGs were stored in a desiccator to maintain dryness and prevent moisture absorption.

### 2.3. Preparation of Bio-Composite Compounds

The raw materials were initially dehydrated in a hot air oven at 70 °C for 4 h to remove any residual moisture. This step was crucial to prevent moisture-related issues during the processing of the composites. Following the dehydration, PLA was blended with exSCGs at varying loadings of 5, 10, and 15 wt% exSCGs. The blending of PLA and exSCGs was carried out using a twin-screw extruder (LABTECH Model LTE16–40) (Labtech Engineering Co. Ltd., Samutprakarn, Thailand), which allowed for consistent mixing and compounding of the materials. The temperature profile for the extrusion process was carefully controlled, ranging from 100 °C at the feed section to 180 °C at the die to ensure the optimal melting and flow characteristics of the PLA while avoiding thermal degradation. The screw speed was set at 100 rpm to achieve efficient mixing without causing shear-induced breakdown of the materials. The composite materials of the PLA/exSCG composites with 5, 10, and 15 wt% exSCGs were extruded into filaments, which were subsequently cooled and cut into pellets for further processing and testing.

### 2.4. Preparation of Bio-Composite Films

The bio-composite films were fabricated using a blow film extruder (LABTECH Model LF-250). (Labtech Engineering Co. Ltd., Samutprakarn, Thailand) Prior to extrusion, the PLA/exSCG composite pellets, prepared as described above, were loaded into the extruder hopper. The extrusion process was carried out with a carefully controlled temperature profile, ranging from 150 °C at the feed section to 180 °C at the die to ensure proper melting and flow of the polymer matrix while preventing degradation of both PLA and exSCGs. The screw speed was set between 80–90 rpm to facilitate optimal mixing and consistent extrusion of the composite material. During extrusion, the polymer was forced through a die and inflated using air pressure to form a thin film. The film thickness was controlled to approximately 60–70 µm, ensuring it was both flexible and suitable for further processing. The final extruded film width was 17 cm, and the films were cooled by ambient air as they were drawn away from the die. The resulting films were collected and cut into appropriate sizes for further analysis or application. The film formation process was carefully monitored to ensure uniformity in thickness, width, and surface quality.

### 2.5. Characterization of Spent Coffee Grounds (SCGs) and Extracted Spent Coffee Grounds (exSCGs)

Particle size distribution: SCG and exSCG particle size distribution was analyzed by a scanning electron microscope (SEM) (Model Leo1455VP, CARL ZEISS Co., Ltd., Oberkochen, Germany). Particle size was reported as an average from 100 measurements of each image (imageJ 1.53t (64-bit)).

Morphology: SCG and exSCG particles, bio-composite compounds, and films (1.00 cm^2^ × 1.00 cm^2^) were analyzed using a scanning electron microscope (SEM) (model Leo1455VP, CARL ZEISS Co., Ltd., Oberkochen, Germany) and field emission scanning electron microscope (FESEM) (model Apero S, ThermoFisher, Waltham, MA, USA) The samples were placed on a stub with carbon tape sputter-coated with gold.

Melt flow index (MFI): Neat PLA and PLA/exSCG bio-composite compounds produced by twin screw extrusion were evaluated for their melt flow rate via a melt flow indexer (Instron^®^ CEAST MF20, Pianezza, TO, Italy.), according to ASTM D1238E standard methodology [32]. Bio-composite compound pellets of approximately 8.00–12.00 g were used, with MFI testing done at 190 °C and a 2.16 kg load.

Capillary flow rheology: The flow properties of neat PLA and PLA/exSCG bio-composite compounds were studied using a capillary rheometer (Rosand RH7, comprehensive rheological analysis from Malvern, Worcestershire, UK) at 180 °C with a shear rate of 50–3000 s^−1^ and sample weight of around 25–30 g per test. Melt strength was measured using an extrusion melt temperature of 180 °C with a capillary die (D = 1 mm, L/D = 16 entrance angle = 90°) and “Tragethon” haul-off (melt strength).

Fourier transform infrared (FTIR) spectroscopy: FTIR spectroscopy was done using a Perkin Elmer model Spectrum GX spectrometer (Hopkinton, MA, USA). All samples were analyzed in ATR mode, scanning wavenumbers from 4000–400 cm^−1^.

Color parameter: Bio-composite films with an area of 15 cm x 15 cm were analyzed in 10 different positions to measure the color parameter, using a colorimeter (Konica Minolta model CR-20, Tokyo, Japan) following ASTM E313-96 standard methodology [33]. Data were reported in a CIELAB system with the color difference (∆E) calculated as follows.

$$∆E=\sqrt{{\left(L_{2}^{*}-L_{1}^{*}\right)}^{2}+{\left(a_{2}^{*}-a_{1}^{*}\right)}^{2}+{\left(b_{2}^{*}-b_{1}^{*}\right)}^{2}}$$

where: ∆L* = Difference in lightness and darkness (+ light, − dark). ∆a* = Difference between red shade and green shade (+as red shade, − as green shade). ∆b* = Difference between yellow shade and blue shade (+as yellow shade, − as blue shade).

UV light barrier: Neat PLA and PLA/exSCG bio-composite films were measured for their % transparency using a UV–vis spectrophotometer (Specord 210 plus, Analytik Jena GmbH+Co. KG, Jena, Germany). Film specimens were cut to a size of 3.00 cm × 3.00 cm, with 5 pieces analyzed per sample. Recordings were done from wavelengths of 200–700 nm with a scanning rate of 50 mm/min, where %T was reported for the UVC region (280 nm), UVB region (315 nm), UVA region (400 nm), and visible region (700 nm). Opacity was expressed as percent transmittance (%T) at 660 nm, where a %T value of < 30% was determined as opaque, %T = 31–45% as semi-translucent, %T = 46–75% as translucent, and %T = 75% as optically clear.

Mechanical properties: Tensile testing was performed using a universal testing machine (INSTRON^®^ CALIBRATION LAB model 5965, Norwood, MA, USA) according to ASTM D882 standard methodology [34]. The specimens were prepared in a dumbbell shape, with 7 specimens analyzed per sample. Testing was done using a 1 kN load cell, a tension rate of 20 mm/min at 25 °C ± 2, and a relative humidity of 50% ± 5. Tensile strength, % elongation at break, and modulus were reported.

Thermogravimetric analysis (TGA): Thermal gravimetric analysis (TGA) was performed (METTLER TOLEDO model DSC/TGA, Greifensee, Switzerland) at a temperature scan from 25–800 °C, with a heating rate of 10 °C/min under a nitrogen atmosphere (flow rate = 30 mL/min). The degradation temperature (T_d_) was determined as the initial step of weight loss.

Differential scanning calorimetry (DSC): Differential scanning calorimetry (METTLER TOLEDO, DSC 1, Greifensee, Switzerland) was performed under a nitrogen atmosphere. Samples of 8.00–12.00 mg were used for analysis. The first heating scan was from 25 °C to 200 °C, at a heating rate of 10 °C/min, and then held at a temperature of 200 °C for 2 min. The subsequent cooling scan was done from 200 °C to 25 °C, at a cooling rate of 10 °C/min. The second heating scan was from 25 °C to 200 °C, at a heating rate of 10 °C/min. The degree of crystallinity (X_c_) was calculated as follows.

$$\%Crystallinity=\frac{{∆H}_{m}}{{∆H}_{m^{*}}\cdot (1-X)}\times 100$$

where: ∆*H_m_* = Enthalpy of the melting peak in endothermal process (J/g). ${∆H}_{m^{*}}$ = Enthalpy of the melting peak of pure PLA (93 J/g). *X* = Weight fraction of filler in the biopolymer.

Biodegradation: The biodegradation of neat PLA and of the PLA/exSCG bio-composite films was done using a soil burial technique under lab control conditions in a test box, using a moisture content of around 70–90%, with water applied every day, at a temperature of 29–35 °C. The test was also done in natural conditions, with a temperature ranging from 24–48 °C and a moisture content of around 70–90%, with water applied every day, in Phitsanulok, Thailand. Film samples were cut into 3.00 cm × 3.00 cm squares with 5 specimens analyzed per test. The biodegradability was assessed through optical observation and SEM analysis of surface morphology over a 9-month period.

Field test: A field test was conducted using nursery bags made from PLA with an exSCG content of 10 wt.%, of 10.16 × 17.78 cm with a diameter of 20.00 cm (this size is referenced from commercial nursery bag that is 4 × 7 inches (folded) and has diameter of 7.8 inches (open)) with film thicknesses of 80, 100, and 120 µm. Each thickness variant included approximately 120 bags, which were tested over a period of three months. These bio-composite nursery bags were used for planting chili and monitored for changes in appearance, color, morphology, and mechanical properties over the testing period.

Statistical analysis: Quantitative data are reported as the mean ± standard deviation (SD) to represent data dispersion and variability within each group. The results are presented descriptively to highlight observed trends and differences in the dataset.

## 3. Results and Discussion

### 3.1. SCGs Before and After Oil Extraction

#### 3.1.1. Physical Properties

**Figure 1:**
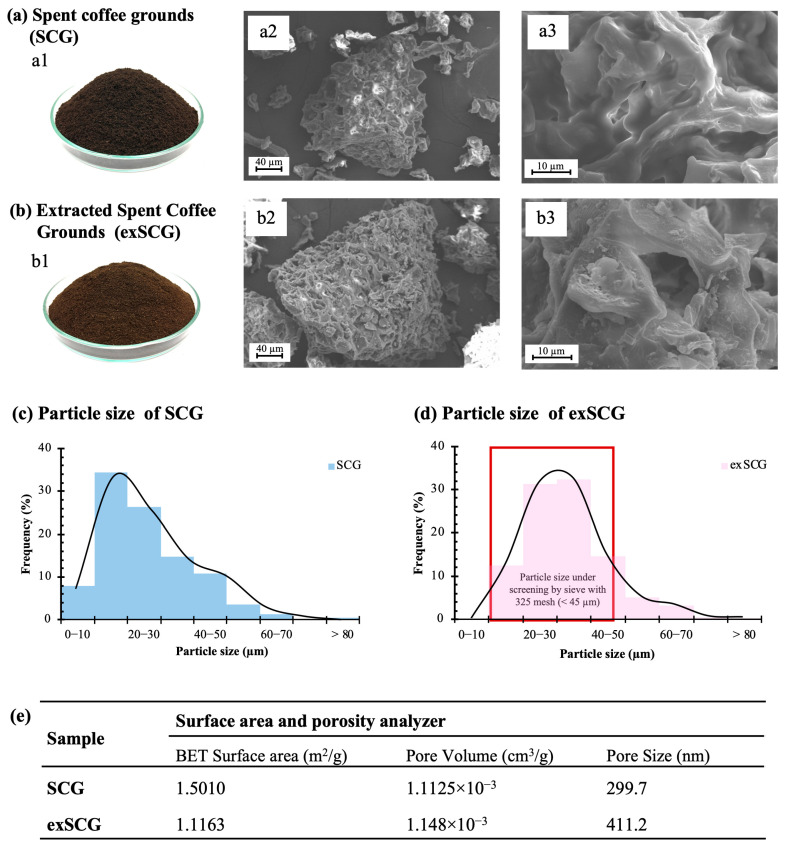
Optical images of SCGs (**a1**) and exSCGs (**b1**). Surface morphology of SCGs (**a2**,**a3**) and exSCGs (**b2**,**b3**) via SEM imaging. Particle size distribution of SCGs (**c**) and exSCGs (**d**) screened through a 325-mesh sieve, and surface area (**e**).

The physical morphology of SCGs both before (SCGs) and after (exSCGs) the oil extraction process was studied, including particle size, surface area, and porosity. SCGs, which contain coffee oil, exhibited a dark brown color (see Figure 1a1), attributed to the organic compounds produced from the Maillard reaction during the seed roasting process [22,35]. A lighter brown color was observed for exSCGs (Figure 1b1). Spent coffee grounds (SCGs) have been reported to contain between 16.7 and 17.2 wt% oil [31]. After the first extraction using hexane, approximately 14 wt% of the oil is removed, followed by an additional 3.46 wt% in a second extraction and 1.13 wt% in a third. In this research, the term “exSCGs” specifically refers to SCGs after the first round of oil extraction. The particle diameter of SCGs is approximately 10–30 µm (Figure 1c), whereas exSCG particles have a larger diameter of approximately 20–40 µm (Figure 1d). The larger size of exSCGs compared to SCGs is due to the sieving process used during the washing of SCGs with water, followed by screening through a 325-mesh (<45 µm) sieve. This process retains larger particles in the exSCGs compared to the initial SCG particles. However, particles larger than 45 µm were still present due to their irregular shape, with some having elongated forms that allow smaller diameters to pass through the sieve while their longer dimensions remain above the 45 µm threshold. SEM images of SCGs (Figure 1a2,a3) and exSCGs (Figure 1b2,b3) reveal that SCGs have a rougher surface compared to exSCGs, attributed to a waxy coating from the coffee oil content. Brunauer–Emmett–Teller (BET) analysis (Figure 1e) shows a significant decrease in surface area after coffee oil extraction, from 1.5010 m^2^/g (SCGs) to 1.1163 m^2^/g (exSCGs), and an increase in pore size from ~300 nm (SCGs) to ~410 nm (exSCGs). The increase in filler pore size significantly influences the biodegradation performance of the bio-composite material by enhancing microbial colonization and water absorption, ultimately leading to an increased rate of biodegradation.

#### 3.1.2. Chemical Functionality

**Figure 2:**
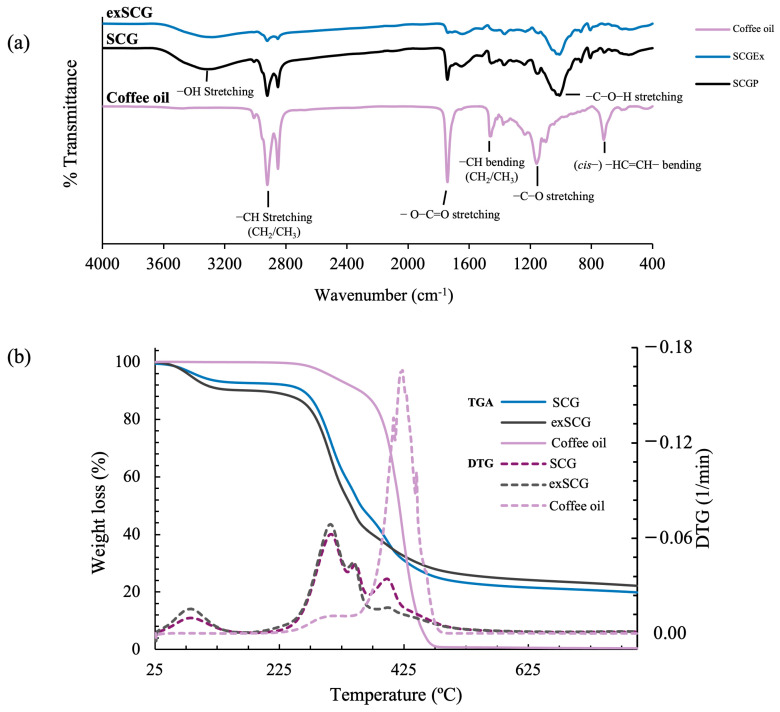
FTIR spectra (**a**) and TGA thermograms (**b**) of spent coffee grounds (SCGs), extracted spent coffee grounds (exSCGs), and coffee oil.

The chemical functionality of SCGs, exSCGs, and coffee oil was analyzed and compared using Fourier transform infrared (FTIR) spectroscopy (Figure 2a). This analysis allowed for the observation of changes in chemical structures associated with the extraction of coffee oil from SCGs to exSCGs, providing insight into the impact of the extraction process on the molecular composition of the materials. Both SCGs and exSCGs exhibited characteristic peaks related to hydroxyl groups (–OH stretching) at 3312 cm^−1^ and 3287 cm^−1^, respectively, which are indicative of the lignocellulosic nature of these materials [22]. Additional peaks observed in the FTIR spectra of SCGs and exSCGs were consistent with those of coffee oil, such as the C–O stretching vibration in the –C–O–H region of glycosidic bonds at 1008 cm^−1^ and 1009 cm^−1^, the (cis)–HC=CH– bending vibration at 720 cm^−1^, and the ester group (O–C=O stretching) at 1742 cm^−1^. These findings suggest that both SCGs and exSCGs still contain residual coffee oil. However, the intensity of these peaks was lower in exSCGs compared to SCGs, indicating that the extraction process effectively removed a significant portion of the coffee oil, although not all of it. This partial removal of coffee oil from exSCGs is beneficial for subsequent blending processes, as the remaining oil could contribute to enhanced material properties.

#### 3.1.3. Thermal Properties of Compounds

Thermal gravimetric analysis (TGA) thermograms for SCGs, exSCGs, and coffee oil are presented in Figure 2b. Both SCGs and exSCGs exhibit similar thermograms with four distinct steps of mass loss. The first step, occurring between 50 °C and 100 °C, represents moisture loss (sample dehydration), with 6.78% and 9.69% mass lost for SCGs and exSCGs, respectively. The second step, observed between 260 °C and 300 °C, corresponds to the depolymerization and decomposition of hemicellulose [36], resulting in mass losses of approximately 33.32% and 37.19% for SCGs and exSCGs, respectively. The decomposition temperature observed between 342 °C and 348 °C corresponds to the breakdown of cellulose, resulting in mass losses of 13.51% and 12.19% [37]. The final step, occurring between 394 °C and 398 °C, involves the decomposition of lignin and fatty acids. This step leads to weight losses of approximately 26.00% and 18.34% for SCGs and exSCGs, respectively [38]. Even at 800 °C, solid residues remain, accounting for 20.39% and 22.59% of the total mass for SCGs and exSCGs, respectively. Furthermore, coffee oil exhibits two distinct weight loss steps. The first step, observed between 250 °C and 300 °C, involves the decomposition of unsaturated fatty acids, resulting in a weight loss of 10.43%. The second step, occurring between 400 °C and 470 °C, involves overlapping oxidative decompositions of remaining unsaturated fatty acids, with saturated fatty acids being predominant in the composition of coffee oil [39]. This indicates the thermal stability of exSCGs, which is essential for evaluating their thermal-oxidative stability during processing, especially when employing extrusion and blow film techniques.

### 3.2. PLA/exSCG Composites

Extracted spent coffee grounds (exSCGs) were incorporated into poly(lactic acid) (PLA) to fabricate composite films through a two-step process involving the production of composite pellets via twin screw extrusion, followed by blown-film extrusion to produce PLA/exSCG composite films. Various compositions of exSCGs were employed in the study to identify the optimal formulation that enhances melt processability by lowering the melt flow index of the PLA/exSCG composites compared to PLA/SCG composites, as reported in previous studies [29]. The primary objective was to improve the physical and mechanical properties of the resulting composite films, ensuring their suitability for agricultural applications, particularly as biodegradable nursery bags. By systematically evaluating the effects of different exSCG concentrations on the melt flow index and the overall performance of the composite films, this study aims to establish a balance between processability and material properties, ultimately contributing to the development of sustainable agricultural solutions.

#### 3.2.1. Melt Flow Index (MFI)

**Figure 3:**
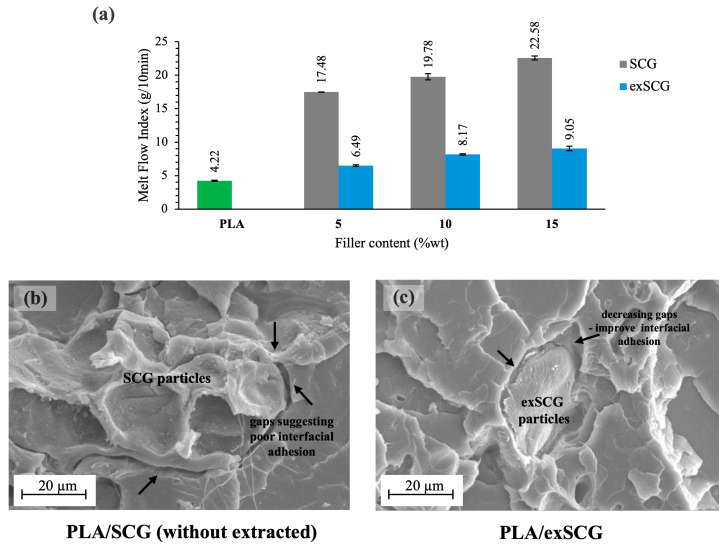
MFI of neat PLA and PLA/exSCGs at various compositions of exSCGs (**a**). SEM images of PLA/SCGs (**b**) and PLA/exSCGs (**c**), highlighting the improvement in interfacial adhesion between PLA and exSCGs when compared to PLA and SCGs.

The melt rheological properties of PLA-based bio-composite compounds, indicated by their melt flow index (MFI), were analyzed with varying exSCG weight contents. Figure 3a presents the MFI values for neat PLA and PLA/exSCG bio-composites, compared to PLA/SCGs. Both bio-composite films exhibited higher melt flow rates (lower melt viscosity) than neat PLA, with PLA/exSCGs showing significantly lower MFI than PLA/SCGs. This demonstrates the effect of SCGs and exSCGs on the melt viscosity of the bio-composites, primarily due to the differing coffee oil contents. A higher coffee oil content results in a higher melt flow rate, likely because the coffee oil migrates out of the particles during the melting process, acting as a plasticizer and enhancing the chain mobility of PLA. This plasticizing effect decreases the melt viscosity, making the material flow more easily. Additionally, the higher oil content likely reduces shear friction within the extruder’s screw barrel, which can hinder the effective mixing of the filler with the polymer matrix. This finding aligns with our previous research, which showed that PLA/SCG bio-composites exhibited higher melt flow rates (PLA/SCGs 10 wt% = 19.71 ± 0.50 g/10 min) compared to neat PLA (4.22 g/10 min) due to the presence of coffee oil [29]. The SEM images (Figure 3b,c) further illustrate that exSCGs achieve better interfacial interaction with the PLA matrix than non-extracted SCGs, likely due to the removal of coffee oil. Additionally, PLA/exSCG bio-composites exhibited MFI values in the range of 6–10 g/10 min, indicating good flowability. This MFI range strikes a balance between processability and material strength, as materials within the 5–15 g/10 min range are known for their ease of processing, stable bubble formation, and better control of film thickness during extrusion [40].

#### 3.2.2. Capillary Rheology

**Figure 4:**
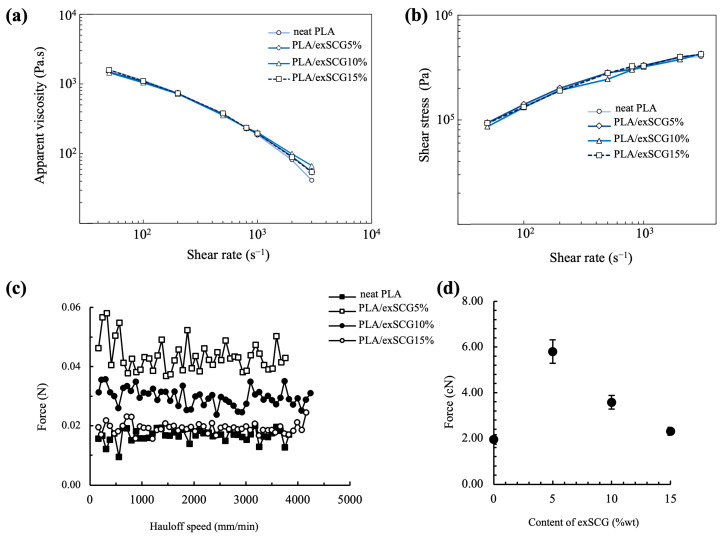
Capillary rheology results for neat PLA and PLA/exSCG bio-composites; apparent viscosity versus shear rate (**a**), Shear stress versus shear rate (**b**), force versus hau-loff speed showing the melt strength (**c**) and melt strength with varying concentration of exSCGs (**d**).

The rheological properties, assessed through capillary rheology of the bio-composite melts, are crucial for blown film extrusion processes. Figure 4a illustrates that the apparent viscosity of both neat PLA and the PLA/exSCG bio-composites decreases as the shear rate increases from 50 s^−1^ to 3000 s^−1^. Notably, the relationship between apparent viscosity and shear rate of neat PLA and PLA/exSCGs was non-linear, exhibiting typical non-Newtonian pseudoplastic behavior [41]. Melt strength represents the maximum tension that can be applied to the polymer melt without rupture or tearing [42]. However, further increases in exSCG content led to a gradual decline in melt strength (Figure 4c,d). At high shear rates, PLA/exSCG exhibits a slight increase in apparent viscosity relative to neat PLA. In particular, PLA/exSCG5% and PLA/exSCG10% demonstrate a marked increase in melt viscosity at high shear rates, rendering it ideally suitable for blown film applications.

### 3.3. PLA/exSCG Bio-Composite Films

#### 3.3.1. Physical Appearance and Surface Morphology

**Figure 5:**
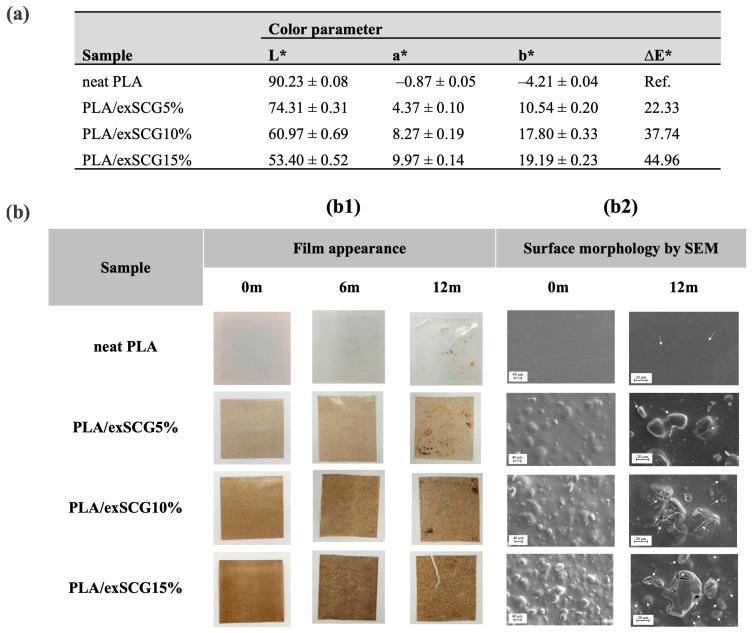
Color parameter of the film (**a**) and physical change of the films after soil burial test (**b**); film appearance by photography (**b1**), and surface morphology via SEM analysis with surface cracking highlighted by white arrows (**b2**), of neat PLA and PLA/exSCG bio-composite films before and after soil burial test.

Photographs and SEM images showing the surface morphology of the bio-composite films with different concentrations of exSCGs are presented, along with the corresponding color parameters (Figure 5). The opacity of neat PLA is 98.68 ± 1.34%, with an L* value of 90.23 ± 0.08, confirming that the neat PLA film is optically clear (%T at 660 nm = 98.65%). Conversely, the bio-composite film with 5–15 wt% exSCGs exhibited a reduction in opacity, leading to opaque characteristics (%T at 660 nm = 11.00%, 2.67%, and 1.00%, respectively). The L* value decreased from 90.23 ± 0.08 to 53.40 ± 0.52 with increasing exSCG content (neat PLA to PLA/exSCG15%), while the a* value increased from –0.87 ± 0.05 to 9.97 ± 0.14, and the b* value increased from –4.21 ± 0.04 to 19.19 ± 0.23, indicating a shift to a light brown color. This color change is attributed to the presence of melanoidin [23], a brown compound formed during the roasting of coffee beans [43]. The surface morphology of neat PLA displayed a smooth finish, whereas the PLA/exSCG film exhibited a rougher surface with well-distributed exSCG particles. Consequently, the opaque character of the PLA/exSCG bio-composite films has allowed for improved light-blocking characteristics, suitable for application as a nursery bag, by helping to protect the contained plant roots from sunlight.

#### 3.3.2. UV Light Barrier Properties

**Figure 6:**
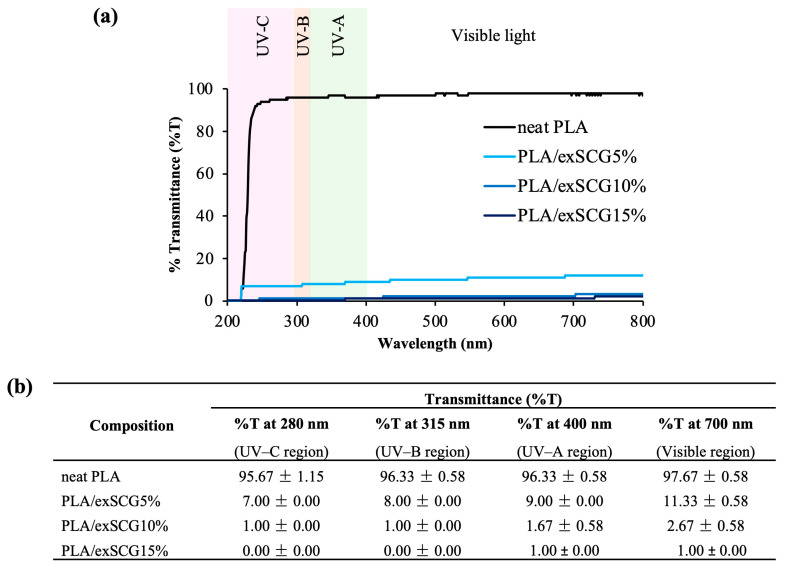
Transmission (%T) curve (**a**) and value (**b**) of the neat PLA and PLA/exSCG bio-composite films.

Various types of UV irradiation, particularly AI1, are known to adversely affect plant processes. Figure 6 displays the UV transmission (%T) curve (Figure 6a) and values (Figure 6b) of the bio-composite films. Neat PLA allows more than 98% UV transmissions, but with the inclusion of exSCG particles, the light barrier properties of the biopolymer films are enhanced. Films with higher exSCG content exhibit the least light transmittance, with PLA/exSCGs15% yielding approximately 1–2% transmittance across the UVC, UVB, UVA, and visible light spectra. Notably, bio-composite films with any exSCG content provide significant protection to plant roots by reducing %T to less than 88%, effectively shielding them from light damage across the UV and visible light range. This impressive UV light absorption performance is attributed to the presence of antioxidant compounds, such as melanoidin or fatty acids in exSCG particles. Melanoidin, a brown-colored compound, derived from the roasting process of coffee beans, is particularly abundant in exSCG particles. Therefore, the opaque nature of PLA/exSCGs makes it well suited for use in biodegradable nursery bags designed to protect plant roots from sunlight exposure [44].

#### 3.3.3. Mechanical Properties

**Figure 7:**
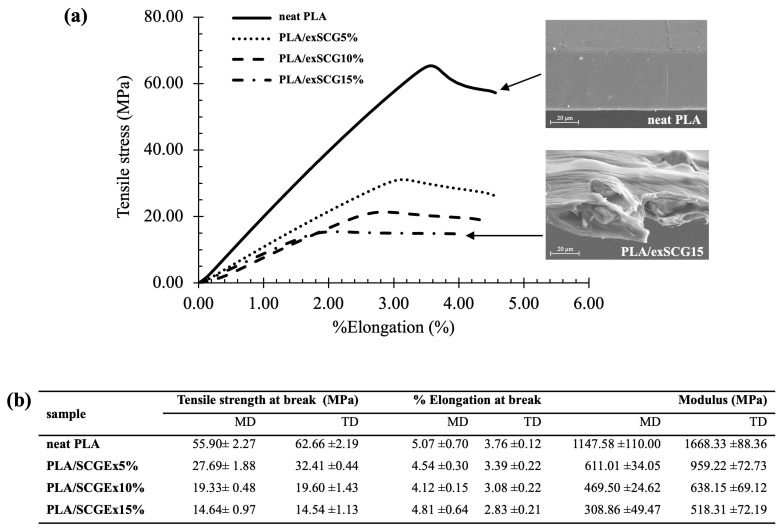
Stress–strain curves (**a**) and tensile mechanical properties (**b**) of neat PLA and the PLA/exSCG bio-composite films.

The mechanical properties of neat PLA and PLA/exSCG bio-composite films are presented in Figure 7b. Films were tested in both the machine direction (MD) and transverse direction (TD), following ASTM D882 standard methodology. As outlined in Figure 7b, the tensile strength results for samples in the transverse direction were similar to the machine direction. The tensile strength at break decreased with increasing exSCG content, with all PLA/exSCG blends exhibiting lower tensile strength compared to neat PLA (55.90–62.66 MPa). This reduction is attributed to the disruption of the PLA chain structure by the exSCG filler. A slight decrease in % elongation at break was also observed in PLA/exSCG blends relative to neat PLA, consistent with the common effect of microparticle addition on the mechanical properties of thermoplastic composites. Notably, the modulus of PLA/exSCG blends decreased by approximately 60% compared to neat PLA, indicating the significant softening of the bio-composite [45]. The decrease in modulus with increasing exSCG content can be attributed to the poor interfacial interactions between the exSCG particles and the PLA matrix. This weak bonding leads to reduced reinforcement from the filler, causing the material to behave more like a softer matrix. SEM images (Figure 7a) confirm this by showing filler-matrix debonding (filler pull-out) under stress, which further weakens the composite structure. Additionally, the stress–strain curves and SEM images of fractured surfaces after tensile testing reveal the influence of residual coffee oil, which acts as a plasticizer. This plasticizing effect likely reduces the stiffness of the PLA matrix, contributing to the observed decrease in modulus and the overall mechanical softening of the PLA/exSCG bio-composite films. Additionally, the tensile strength of the PLA/exSCG composite films exceeds the minimum requirement of 10 MPa specified by Thai Standard 2996-2562 [46] for biodegradable nursery bags. Furthermore, the softening behavior of PLA/exSCGs enhances its functionality, making it more user-friendly and practical for application.

#### 3.3.4. Thermal Properties

**Figure 8:**
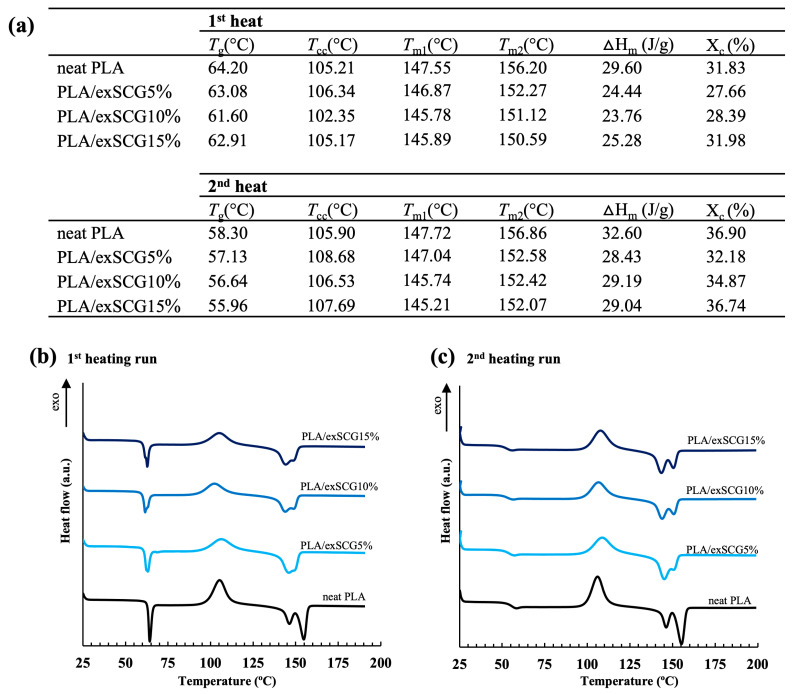
Thermal properties (**a**) and DSC thermograms of the first (**b**) and second heating runs (**c**) of neat PLA and PLA/exSCG bio-composite films.

Thermal properties were measured by DSC analysis, with the glass-transition temperature (*T*_g_), melting temperature (*T*_m_), cold crystallization temperature (*T*_cc_), melting enthalpy (Δ*H_m_*), cold crystallization enthalpy (ΔH_cc_), and degree of crystallinity (X_c_) for the first heat and second heat scans displayed in Figure 8b. For the first heat scan, the *T*_g_ decreased from 64 °C (neat PLA) to 61 °C (PLA/exSCGs15%) with the addition of exSCGs. The exSCGs act as a micrometric filler, with residual coffee oil released at high shear stresses during the processing step and thus able to directly interact with polymer chains and therefore increase the free volume and chain mobility. Notably, the degree of crystallinity of neat PLA is 31.83%, but with the addition of 5 wt% exSCGs, the degree of crystallinity decreased to 27.66%, followed by an increase to 28.39% and 31.98%, with 10 wt% and 15 wt% exSCG content, respectively (Figure 8a). In the second heating step, the *T_g_* of neat PLA decreased from 58.30 °C to 55.96 °C with the incorporation of exSCGs in the PLA/exSCG bio-composite. At higher exSCG content, the melting temperature (*T*_m_) of PLA/exSCGs shifted to lower temperatures, accompanied by a reduction in crystallinity (%*X*) (Figure 8a). This suggests that the coffee oil in exSCGs acts as a plasticizer, increasing the chain mobility of PLA. The plasticizing effect disrupts the polymer’s ability to crystallize, leading to lower crystallinity and, consequently, a reduced melting temperature. This behavior is consistent with the weakening of intermolecular forces and the impaired formation of crystalline regions within the PLA matrix.

#### 3.3.5. Biodegradation Under Lab Conditions (Soil Burial)

Neat PLA and PLA/exSCG bio-composite films underwent biodegradability testing under laboratory conditions in a dark box, with the soil moisture maintained at 70–90% relative humidity (RH) and temperatures ranging from 29–35 °C. The biodegradation of both neat PLA and PLA/exSCG bio-composite films is primarily reflected in changes to the surface morphology. The degradation of PLA, conducted at temperatures below its glass transition temperature (T_g_), proceeds slowly via hydrolytic mechanisms [47]. After 12 months of soil burial (Figure 5b1), neat PLA displayed only slight surface roughening with no major physical changes. In contrast, the PLA/exSCG bio-composite film showed visible cracks, indicating a significant loss in mechanical integrity. SEM images (Figure 5b2) further confirm increased surface roughness and crack formation, particularly around the exSCG regions. The faster degradation of the PLA/exSCG bio-composite can be attributed to the water absorption of exSCG particles, which causes them to swell and weaken the polymer matrix, promoting crack formation. Additionally, the polysaccharides and fatty acids in exSCGs are highly susceptible to microbial degradation in high-moisture environments, accelerating the breakdown of the composite. This enhanced biodegradability is supported by the increased water retention in the bio-composite, which fosters microbial activity and accelerates the overall degradation process [48,49].

#### 3.3.6. Biodegradation Field Test

The biodegradation field test aims to evaluate the environmental performance of the bio-composite film PLA/exSCG10% under real-world conditions. This film was chosen for field testing due to its favorable rheological flow behavior, which is critical for large-scale production, as well as its suitable mechanical properties, making it ideal for nursery bags designed to hold soil loads. Additionally, PLA/exSCG10% has shown accelerated degradation in soil compared to neat PLA, highlighting its potential as an eco-friendly solution for agricultural applications. Therefore, PLA/exSCG10% films with varying thicknesses (ranging from 60 µm to 120 µm) were evaluated under natural soil burial conditions. This test aims to investigate the thickness-dependent properties of PLA/exSCG composite films as nursery bags for planting applications. The primary focus was to assess their suitability across a complete plant growth cycle and determine whether the composite films could maintain structural integrity under real planting conditions before undergoing degradation.

**Figure 9:**
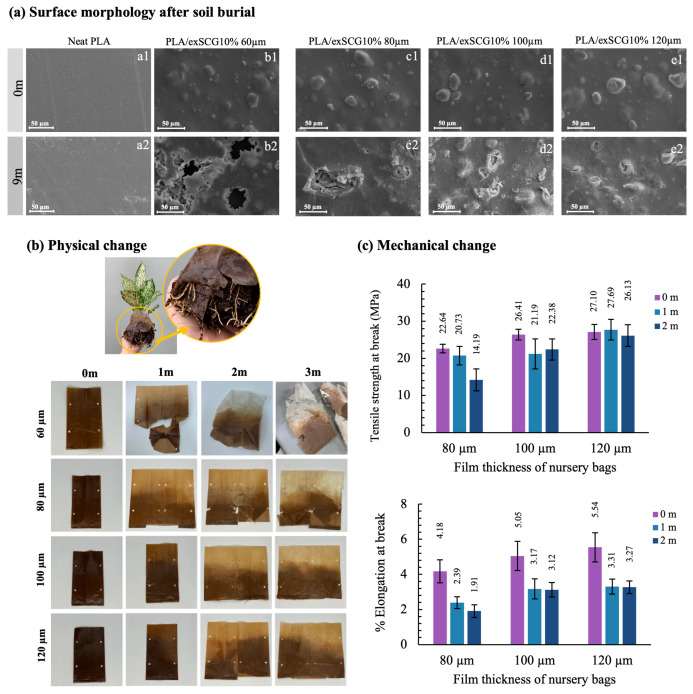
SEM images of neat PLA and bio-composite films during natural soil burial tests at 0 and 9 months (**a**), appearance of the nursery bag showing plant roots emerging from the degraded bag (**b-top**) and the nursery bags with different thickness and field test times (**b-bottom**), and tensile mechanical properties (**c**) of PLA/exSCG10% nursery bags of varying thicknesses after field tests.

Figure 9a presents the SEM images of neat PLA film and PLA/exSCG10% bio-composite films with thicknesses ranging from 60 µm to 120 µm, both at the start (0 months) and after 9 months of soil burial. This field test was conducted under environmental conditions in Thailand’s winter (December–March), with temperatures ranging from 24 °C to 33 °C and relative humidity between 40% and 60%. At the start, the neat PLA film (60 µm) (Figure 9a1) exhibited a clear and smooth surface. After 9 months of soil burial (Figure 9a2), the surface of the PLA film showed increased roughness, but no holes or significant degradation was observed. In contrast, the 60 µm PLA/exSCG10% bio-composite film (Figure 9b1) displayed initial surface roughness due to the distribution of exSCG particles. After 9 months in soil (Figure 9b2), the film was significantly fragmented, with visible holes and surface erosion. This rapid degradation is attributed to the high cellulose and hemicellulose content in the exSCG particles, which promote microbial and insect activity, accelerating the breakdown of the material [50]. The PLA/exSCG10% bio-composite films with thicker layers (80, 100, and 120 µm) exhibited slower degradation. The faster degradation of the 60 µm bio-composite film can be attributed to its thinner structure, which allows for greater microbial access and interaction with the surface. The higher surface area-to-volume ratio in thinner films means more exposure to environmental factors such as microorganisms, moisture, and soil components, leading to accelerated biodegradation. Conversely, thicker films have a lower surface area-to-volume ratio, reducing exposure to these degrading factors. As a result, microbial colonization and enzymatic breakdown are slower in thicker films. Additionally, the inner layers of thicker films remain relatively protected from degradation, as microbial activity primarily targets the outer surfaces. Notably, the bio-composite films at thicknesses of 80, 100, and 120 µm exhibited significantly weaker mechanical properties by hand compared to neat PLA, likely due to the degradation they underwent during the 9-month soil burial test.

Figure 9b presents the appearance of the bio-composite bags with varying film thicknesses, where color fading is observed at the upper portions, closer to the soil surface. These portions are exposed to increased sunlight, particularly UVB wavelengths, initiating a degradation process, wherein the solar energy causes polymer chain scission [51]. This results in noticeable color fading and a reduction in the mechanical properties of the bio-composite film. Additionally, the lower parts of the bags are prone to small cracks due to environmental soil conditions, such as high moisture content, elevated temperatures, and root growth.

As illustrated in Figure 9c, the mechanical integrity of the films diminishes as a result of biodegradation. Specifically, the % elongation at break of the bio-composite film with a thickness of 80 µm showed a significant 50% reduction over two months, whereas bio-composite bags with thicknesses of 100 µm and 120 µm demonstrated more stability in the field. However, these bags still exhibited a significant decline in % elongation at break, at approximately 40%, with visible cracking developed in the folded areas of the bags. By the three-month period, all bags could not produce dumbbell-shaped samples for tensile testing due to cracking, signifying their effective biodegradability.

## 4. Conclusions

This study developed biodegradable nursery bags from PLA and extracted spent coffee grounds (exSCGs) using twin screw and blown film extrusion. The inclusion of oil-extracted exSCGs improved flexibility, flowability, and biodegradability, with residual coffee oil acting as a natural plasticizer. The composite exhibited excellent UV light barrier properties, reducing light transmission by over 98%. While higher exSCG content slightly decreased tensile strength, it enhanced biodegradability, as shown by soil burial tests. The PLA/exSCG10% composite demonstrated optimal properties for large-scale production and rapid degradation in field tests, maintaining integrity for ~4 months. These results highlight the potential of PLA/exSCGs biodegradable nursery bags as a sustainable solution for agricultural applications, with opportunities to optimize exSCG content for an ideal balance between mechanical performance and biodegradability. This innovation provides a sustainable solution for reducing microplastic contamination while simultaneously valorizing coffee industry waste. Moreover, the composite aligns with circular economy principles, promoting a closed-loop, waste-free production system.

## Data Availability

The raw/processed data required to reproduce these findings cannot be shared at this time as the data also forms part of an ongoing study.

## References

[1] Peng X., Chen M., Chen S., Dasgupta S., Xu H., Ta K., Du M., Li J., Bai S. (2018). Microplastics contaminate the deepest part of the world’s ocean. Geochem. Perspect. Lett..

[2] Leslie H.A., van Velzen M.J.M., Brandsma S.H., Vethaak A.D., Garcia-Vallejo J.J., Lamoree M.H. (2022). Discovery and quantification of plastic particle pollution in human blood. Environ. Int..

[3] Ragusa A., Svelato A., Santacroce C., Catalano P., Notarstefano V., Carnevali O., Papa F., Rongioletti M.C.A., Baiocco F., Giorgini E. (2021). Plasticenta: First Evidence of Microplastics in Human Placenta.

[4] Stoett P., Scrich V.M., Elliff C.I., Andrade M.M., de M. Grill N., Turra A. (2024). Global Plastic Pollution, Sustainable Development, and Plastic Justice.

[5] Agriculture Grow Bags Market Size, Share, Industry, Forecast and Outlook (2024–2031). www.datamintelligence.com.

[6] Grow Bags VS. Plastic Pots. Bootstrap Farmer. https://www.bootstrapfarmer.com/blogs/grow-bags/grow-bags-vs-pots-advantages-of-grow-bags.

[7] Kyrikou I., Briassoulis D. (2007). Biodegradation of agricultural plastic films: A critical review. J. Polym. Environ..

[8] Ross S., Mahasaranon S., Ross G.M. (2015). Ternary polymer blends based on poly(lactic acid): Effect of stereo-regularity and molecular weight. J. Appl. Polym. Sci..

[9] Tuancharoensri N., Kongprayoon A., Mahasaranon S., Pratumshat S., Viyoch J., Petrot N., Ruanthong W., Punyodom W., Topham P.D., Tighe B.J. (2023). In Situ Compatibilized Blends of PLA/PCL/CAB Melt-Blown Films with High Elongation: Investigation of Miscibility, Morphology, Crystallinity and Modelling. Polymers.

[10] Tuancharoensri N., Ross G.M., Mahasaranon S., Topham P.D., Ross S. (2017). Ternary blend nanofibres of poly(lactic acid), polycaprolactone and cellulose acetate butyrate for skin tissue scaffolds: Influence of blend ratio and polycaprolactone molecular mass on miscibility, morphology, crystallinity and thermal properties. Polym. Int..

[11] Ross S., Topham P.D., Tighe B.J. (2014). Identification of optically clear regions of ternary polymer blends using a novel rapid screening method. Polym. Int..

[12] Bandopadhyay S., Martin-Closas L., Pelacho A.M., DeBruyn J.M. (2018). Biodegradable plastic mulch films: Impacts on soil microbial communities and ecosystem functions. Front. Microbiol..

[13] Suthapakti K., Molloy R., Punyodom W., Nalampang K., Leejarkpai T., Topham P.D., Tighe B.J. (2018). Biodegradable Compatibilized Poly(l-lactide)/Thermoplastic Polyurethane Blends: Design, Preparation and Property Testing. J. Polym. Environ..

[14] Chaiphut M., Ross S., Ross G., Suphrom N., Mahasaranon S. (2021). Influence of the lemongrass powder and polybutylene succinate on the properties of biocomposite films based on poly (lactic acid). Mater. Today Proc..

[15] Sung S.H., Chang Y., Han J. (2017). Development of polylactic acid nanocomposite films reinforced with cellulose nanocrystals derived from coffee silverskin. Carbohydr. Polym..

[16] Waisarikit A., Ross S., Ross G.M., Mahasaranon S. (2023). The influence of cassava starch on the properties of PLA/PBS/SCG films for packaging applications. Polym.-Plast. Technol. Mater..

[17] Coffee—Worldwide|Statista Market Forecast. https://www.statista.com/outlook/cmo/hot-drinks/coffee/worldwide.

[18] Scully D., Jaiswal A., Abu-Ghannam N. (2016). An Investigation into Spent Coffee Waste as a Renewable Source of Bioactive Compounds and Industrially Important Sugars. Bioengineering.

[19] Vardon D.R., Moser B.R., Zheng W., Witkin K., Evangelista R.L., Strathmann T.J., Rajagopalan K., Sharma B.K. (2013). Complete utilization of spent coffee grounds to produce biodiesel, bio-oil, and biochar. ACS Sustain. Chem. Eng..

[20] Kanai N., Honda T., Yoshihara N., Oyama T., Naito A., Ueda K., Kawamura I. (2020). Structural characterization of cellulose nanofibers isolated from spent coffee grounds and their composite films with poly(vinyl alcohol): A new non-wood source. Cellulose.

[21] De Bomfim A.S.C., de Oliveira D.M., Voorwald H.J.C., Coelho de Carvalho Benini K.C., Dumont M.J., Rodrigue D. (2022). Valorization of Spent Coffee Grounds as Precursors for Biopolymers and Composite Production. Polymers.

[22] Ballesteros L.F., Teixeira J.A., Mussatto S.I. (2014). Chemical, Functional, and Structural Properties of Spent Coffee Grounds and Coffee Silverskin. Food Bioproc. Tech..

[23] Mussatto S.I., Machado E.M.S., Martins S., Teixeira J.A. (2011). Production, Composition, and Application of Coffee and Its Industrial Residues. Food and Bioprocess Technology.

[24] Mendes J.F., Martins J.T., Manrich A., Luchesi B.R., Dantas A.P.S., Vanderlei R.M., Martins M.A. (2021). Thermo-physical and mechanical characteristics of composites based on high-density polyethylene (HDPE) e spent coffee grounds (SCG). J. Polym. Environ..

[25] Sohn J.S., Ryu Y., Yun C.S., Zhu K., Cha S.W. (2019). Extrusion compounding process for the development of eco-friendly SCG/PP composite pellets. Sustainability.

[26] Lule Z.C., Kim J. (2021). Properties of economical and eco-friendly polybutylene adipate terephthalate composites loaded with surface treated coffee husk. Compos. Part A Appl. Sci. Manuf..

[27] Nguyen T.A., Nguyen Q.T. (2021). Hybrid Biocomposites Based on Used Coffee Grounds and Epoxy Resin: Mechanical Properties and Fire Resistance. Int. J. Chem. Eng..

[28] Waisarikit A., Ross S., Ross G.M., Udee N., Mahasaranon S. (2021). Materials Today: Proceedings Modified natural rubber glove with spent coffee grounds for prothesis arm cover. Mater. Today Proc..

[29] Suaduang N., Ross S., Ross G.M., Wangsoub S., Mahasaranon S. (2019). The physical and mechanical properties of biocomposite films composed of poly (lactic acid) with spent coffee grounds. Key Eng. Mater..

[30] Suaduang N., Ross S., Ross G.M., Pratumshat S., Mahasaranon S. (2019). Effect of spent coffee grounds filler on the physical and mechanical properties of poly(lactic acid) bio-composite films. Mater. Today Proc..

[31] Ravindranath R., Yousuf R., Khan A., Reddy T.O., Thirumala S.D., Reddy B.R. (1972). Composition and Characteristics of Indian Coffee Bean, Spent Grounds and Oil. J. Sci. Food Agric..

[32] (2020). Standard Test Method for Melt Flow Rates of Thermoplastics by Extrusion Plastometer.

[33] (2020). Standard Practice for Calculating Yellowness and Whiteness Indices from Instrumentally Measured Color Coordinates.

[34] (2012). Standard Test Method for Tensile Properties of Thin Plastic Sheeting.

[35] Zhang H., Zhang H., Troise A.D., Fogliano V. (2019). Melanoidins from Coffee, Cocoa, and Bread Are Able to Scavenge α-Dicarbonyl Compounds under Simulated Physiological Conditions. J. Agric. Food Chem..

[36] García-García D., Carbonell A., Samper M.D., García-Sanoguera D., Balart R. (2015). Green composites based on polypropylene matrix and hydrophobized spend coffee ground (SCG) powder. Compos. B Eng..

[37] Essabir H., Raji M., Laaziz S.A., Rodrique D., Bouhfid R., el kacem Qaiss A. (2018). Thermo-mechanical performances of polypropylene biocomposites based on untreated, treated and compatibilized spent coffee grounds. Compos. B Eng..

[38] Yang H., Yan R., Chen H., Lee D.H., Zheng C. (2007). Characteristics of hemicellulose, cellulose and lignin pyrolysis. Fuel.

[39] Raba D.N., Chambre D.R., Copolovici D.M., Moldovan C., Copolovici L.O. (2018). The influence of high-temperature heating on composition and thermo-oxidative stability of the oil extracted from Arabica coffee beans. PLoS ONE.

[40] Mount E.M., Wagner J.F., Giles H., Haber E.M. (2007). Extrusion Additives.

[41] Dean K.M., Petinakis E., Meure S., Yu L., Chryss A. (2012). Melt Strength and Rheological Properties of Biodegradable Poly(Lactic Aacid) Modified via Alkyl Radical-Based Reactive Extrusion Processes. J. Polym. Environ..

[42] Vlachopoulos J., Polychronopoulos N., Kontopoulou M., Kontopoulou M. (2012). Basic concepts in polymer melt rheology and their importance in processing. Applied Polymer Rheology: Polymeric Fluids with Industrial Applications.

[43] Capek P., Matulová M., Navarini L., Suggi-Liverani F. (2010). Structural features of an arabinogalactan-protein isolated from instant coffee powder of Coffea arabica beans. Carbohydr. Polym..

[44] Cabrera J., Conesa C.M., del Pozo J.C. (2022). May the Dark be with Roots: A Perspective on How Root Illumination May Bias In Vitro Research on Plant–Environment Interactions.

[45] Finkenstadt V.L., Liu C.K., Evangelista R., Liu L., Cermak S.C., Hojilla-Evangelista M., Willett J.L. (2007). Poly(lactic acid) green composites using oilseed coproducts as fillers. Ind. Crops Prod..

[46] (2019). Biodegradable Plastics Nursery Bags.

[47] Fogašová M., Figalla S., Danišová L., Medlenová E., Hlaváčiková S., Vanovčanová Z., Omaníková L., Horváth V., Mikolajová M., Kadlečková M. (2022). PLA/PHB-Based Materials Fully Biodegradable under Both Industrial and Home-Composting Conditions. Polymers.

[48] Palai B., Mohanty S., Nayak S.K. (2021). A Comparison on Biodegradation Behaviour of Polylactic Acid (PLA) Based Blown Films by Incorporating Thermoplasticized Starch (TPS) and Poly (Butylene Succinate-co-Adipate) (PBSA) Biopolymer in Soil. J. Polym. Environ..

[49] Barragán D.H., Pelacho A.M., Martin-Closas L. (2016). Degradation of agricultural biodegradable plastics in the soil under laboratory conditions. Soil Res..

[50] Kumar A., Kalleshwaraswamy C.M., Sharma R., Sharma P., Poonia A. (2022). Biodegradation of Plastic Using Termites and their Gut Microbiota: A Mini Review. IOP Conf. Ser. Earth Environ. Sci..

[51] Andreia A., Gabriela L.B., Manuela S., Vera M.A. (2013). UV Stability of Poly(Lactic Acid) Nanocomposites. J. Mater. Sci. Eng. B.

